# Modified socket shield technique versus unassisted socket healing: A randomized controlled clinical trial using CBCT-based dimensional analysis

**DOI:** 10.4317/medoral.27936

**Published:** 2026-03-07

**Authors:** Luis Miguel Sáez-Alcaide, Carlos Cobo-Vázquez, Jorge Cortés-Bretón-Brinkmann, Luis Sánchez-Labrador, Rosa María López-Pintor, Juan López-Quiles, Jesús Torres García-Denche

**Affiliations:** 1 Department of Dental Clinical Specialties, Faculty of Dentistry, Complutense University of Madrid, Spain

## Abstract

**Background:**

Tooth loss leads to alveolar bone resorption due to deficiency of blood supply and socket shield technique was introduced to prevent this bone loss. Therefore, this study aimed to evaluate the effect of the modified socket shield technique (mSST) without immediate implant placement (test group) compared with unassisted socket healing (USH) (control group) on vertical and horizontal bone dimensional changes.

**Material and Methods:**

Patients requiring dental extraction at a non-molar site were recruited and randomly allocated to the test or control group. Cone-beam computed tomography (CBCT) scans obtained before and 4 months after surgery were superimposed to assess horizontal ridge width and vertical bone height changes. Group comparisons were performed using analysis of covariance (ANCOVA).

**Results:**

Significant differences between the groups were found for buccal height (BH) reduction (p&lt;0.001), buccal bone width (BW) reduction at 1mm, 3mm and 5mm (p&lt;0.001), and overall bone resorption (p&lt;0.001). The test group showed reduced dimensional changes, with mean differences of 1.527mm in BH, 0.982mm in BW1, 0.783mm in BW3, 0.545mm in BW5, and 26.2mm³ in volume resorption (p&lt;0.001). No significant differences were observed in vertical or horizontal bone resorption on the lingual side. Regarding the influence of buccal bone thickness (BBT) on buccal socket dimensional changes, significant differences between groups were detected. A critical threshold of 1.5mm BBT was identified.

**Conclusions:**

The modified socket shield technique without immediate implant placement appears to be a protective factor against post-extraction bone remodeling, particularly in sites with buccal bone thickness less than 1.5mm.

## Introduction

Following tooth extraction, the alveolar bone undergoes rapid remodeling, with approximately 50% of the ridge width being resorbed, primarily at the expense of the buccal bone. This resorption results from the loss of the bundle bone-periodontal ligament (BB-PDL) complex (1). The process is closely associated with the crucial role of the periodontal ligament (PL) in regulating bone remodeling (2). The PL provides essential vascular support to the alveolar bone, forming the structure known as "bundle bone." Consequently, when a tooth is lost, disruption of this complex is unavoidable, leading to subsequent alveolar bone resorption (3). The extent of volumetric loss is difficult to predict, and significant esthetic discrepancies at the gingival margin may arise (4).

To mitigate these alterations, alveolar ridge preservation (ARP) procedures have been proposed and have shown benefits in maintaining ridge volume following extraction (5). However, ARP using bone grafts or soft tissue grafts can only partially compensate for, but not completely prevent, the physiologic resorption process (6). As early as the 1970s, the possibility of retaining roots of endodontically treated teeth for esthetic purposes-specifically to preserve ridge volume and prevent post-extraction alveolar collapse-was introduced, with reports demonstrating favorable outcomes attributed to the preservation of both hard and soft tissues (7 - 10).

Building on this rationale, Hürzeler et al. described the "Socket Shield" (SS) technique in 2010, aiming to preserve a thin buccal fragment of the tooth root to maintain the PL on the buccal aspect of the implant (11). Recent evidence indicates that the SS technique can effectively preserve alveolar bone and maintain marginal bone stability while yielding predictable esthetic outcomes (12 , 13). Although originally performed in conjunction with immediate implant placement (IIP), clinical scenarios exist in which ridge preservation is necessary, but IIP is contraindicated due to insufficient primary stability or inadequate bone anchorage (14).

For such cases, a modification of the original protocol-the modified socket shield technique (mSST)-has been proposed (15). This approach is performed in two stages: first socket shield technique is prepared and implant placement is carried out after 4 to 6 months, once adequate healing has occurred. However, to the authors' knowledge, evidence comparing mSST without immediate implant placement to spontaneous socket healing remains limited.

Therefore, the aim of this randomized clinical trial was to radiologically evaluate the effect of mSST used for ARP, without immediate implant placement, in comparison with unassisted socket healing (USH) in terms of bone dimensional changes at 4 months post-extraction. The primary outcome was to assess horizontal and vertical dimensional changes, and the secondary outcome was to evaluate the influence of buccal bone thickness on these alterations.

## Material and Methods

Study Design

This parallel-arm randomized controlled clinical trial was conducted in accordance with the principles of the Declaration of Helsinki and received approval from the local Ethics Committee (Hospital Clínico San Carlos, Madrid, Spain; REF: 14-034, 24/07/2021). The manuscript follows the CONSORT guidelines (http://www.consort-statement.org/). The study was registered in the ClinicalTrials.gov database under protocol number 21/028-EC_X (ID: NCT05240417).

Sample Size Calculation

Sample size was calculated based on the primary outcome variable (volume of bone resorption) reported in the study by Ávila-Ortiz et al. (2020) (16), whose design and methodology closely resemble those of the present investigation. Using G*Power 3.1 software (Düsseldorf, Germany), and assuming an alpha error of 0.05 and a statistical power of 80%, the estimated required sample size was 8 patients per group.

Population

Participants were recruited through consecutive case sampling among patients attending the Postgraduate Clinic of Oral Surgery at the Complutense University of Madrid (Spain) between September 2022 and July 2023. All patients provided written informed consent prior to inclusion in the study.

Participant Eligibility Criteria

Inclusion Criteria

- Adult patients (&gt;18 years old) requiring extraction of a single-rooted upper or lower tooth (incisor, canine, or premolar) due to caries, fracture, or prosthetic reasons.

- Presence of at least one natural tooth adjacent to the extraction site (mesially and/or distally).

- Presence of an intact socket as assessed on the preoperative CBCT and confirmed following flapless extraction.

- Systemically healthy individuals classified as ASA I or ASA II according to the American Society of Anesthesiologists, and smoking fewer than 10 cigarettes per day.

- Periodontally healthy patients with adequate oral hygiene (bleeding on probing &lt;20%, plaque index &lt;20%).

Exclusion Criteria

- Uncontrolled diabetes (HbA1c &gt;7), osteoporosis, or any systemic or local condition that could compromise postoperative healing.

- History of malignancy or prior radiotherapy or chemotherapy for cancer treatment.

- Pregnancy, intent to become pregnant, or breastfeeding at the time of enrollment.

- Presence of acute infection at the socket site or active periodontal disease.

Randomization and allocation concealment

Randomization was performed by an external investigator (L.S.L.) who did not participate in any of the surgical procedures. After confirmation that patients met the inclusion criteria each participant was assigned a study identification number, and a corresponding randomization code was generated using dedicated software (GraphPad Software Inc., La Jolla, CA), ensuring double blinding. A restricted randomization method was then applied to determine the intervention allocated to each patient.

Radiological outcome assessment was conducted by independent, calibrated examiners (C.CV. and J.L.Q) who were not involved in the surgical procedures or randomization process. Prior to analysis, all CBCT datasets were anonymized and assigned a numerical code by an independent investigator (J.C.B.B). The evaluators had no access to treatment allocation and remained blinded throughout the entire measurement process.

Treatment Procedures / Interventions

Before the surgical procedure, all patients underwent clinical examination, clinical photography, panoramic radiography, and cone-beam computed tomography (CBCT1) to confirm eligibility. In addition, all patients received a periodontal evaluation and individualized oral hygiene instructions to optimize oral conditions before treatment.

All surgical procedures were performed by a single oral surgeon (L.M.S.) under local anesthesia at the Postgraduate Clinic of Oral Surgery, Complutense University of Madrid (Spain). This surgeon was not involved in any of the outcome assessments.

**Figure 1:**
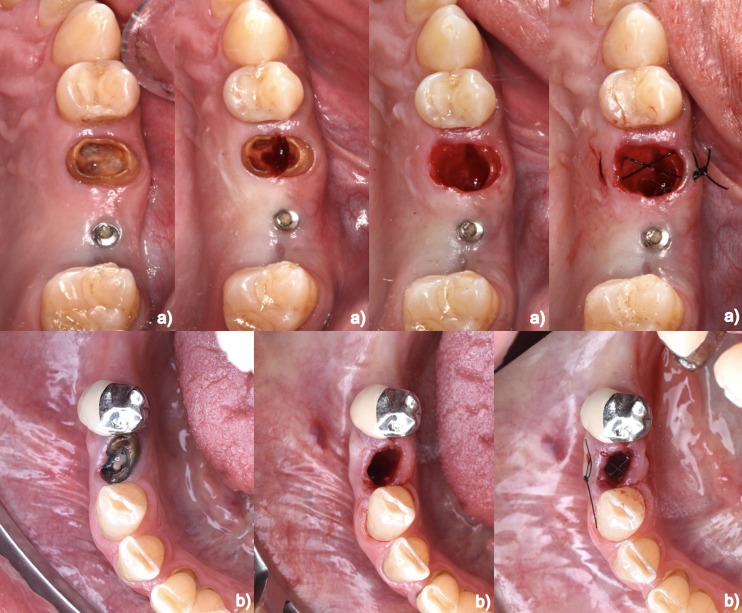
Step by step description of the treatment procedures: a) experimental group; b) control group.

- Control group (USH): Atraumatic, flapless tooth extraction was performed using forceps and fine elevators when necessary.

- Experimental group (mSST): The modified socket shield technique was performed according to a modification of the protocol described by Glocker in 2014 (15):

- The root was longitudinally sectioned in the mesiodistal direction as apically as possible using a long-shank root resection bur (Komet Dental).

- A periotome was then used to carefully remove the palatal fragment while avoiding any movement of the buccal portion.

- The remaining buccal root fragment was reduced coronally to approximately 1 mm above the alveolar crest, contoured, and thinned using a long-shank round diamond bur (Komet Dental).

- The socket was thoroughly curetted to remove all soft tissue remnants, irrigated with sterile saline, and sealed with a single crossed mattress suture using 5/0 nylon (Figure 1).

1All patients received systemic antibiotics (amoxicillin 500mg, three times daily for 5 days), analgesics as required (ibuprofen 600mg), and were instructed to rinse twice daily for 10 days with 0.2% chlorhexidine (PerioAid®, Dentaid, Spain). Patients were recalled at 10 days for suture removal, during which clinical photographs were taken and any complications were documented. A follow-up appointment was scheduled at 4 months postoperatively.

Radiological evaluation

Before the surgical procedures, a cone-beam computed tomography (CBCT1) scan was obtained using a CBCT unit (CS 8100 3D®, Carestream Dental DLL). A second CBCT scan (CBCT2) was performed 4 months postoperatively. All scans were acquired using a standardized acquisition protocol with a cylindrical field of view (FOV) of 8×8cm and a voxel size of 0.16 mm, following the manufacturer's recommended settings (scan time: 15s; exposure time: 1.8s).

Computer-assisted superimposition of the CBCT datasets was performed using 3D Slicer® software by an independent and calibrated examiner with more than 5 years of experience in digital DICOM file superimposition (J.T.G.).

Baseline buccal bone thickness (BBT) was measured in CBCT1 to assess its possible relationship with subsequent dimensional changes.

Following the methodology proposed by Jung et in 2013 (17), two independent and calibrated researchers (C.C.V. and J.L.Q) performed the radiological assessment. Measurements were initially conducted on the baseline CBCT using the distance to the mesial and distal bone ridges in the axial section, after which a cross-sectional image through the center of the involved tooth was selected. The CBCT obtained at 4 months was analyzed using the same procedure. Vertical and horizontal reference lines were drawn as described by Jung et al (17).

**Figure 2:**
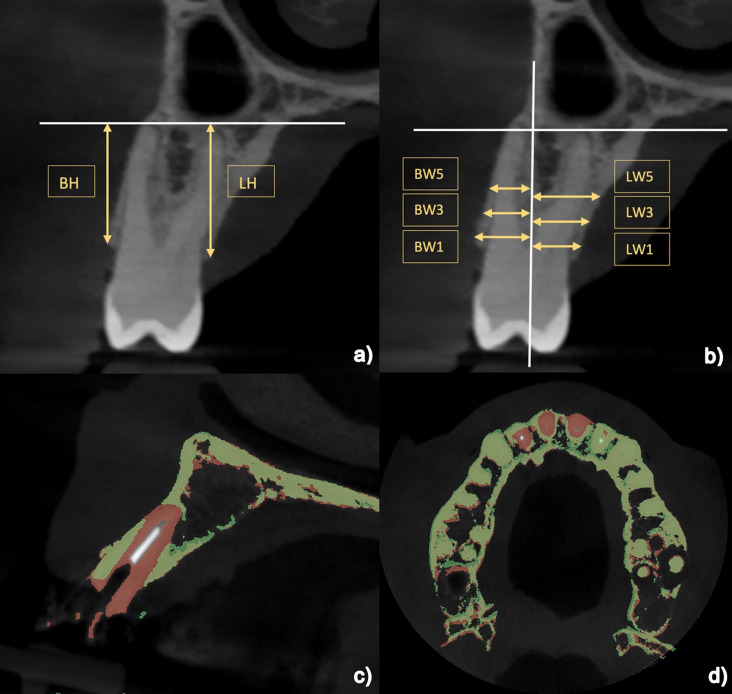
Radiographic measurements on CBCT: a) Vertical references of the buccal and lingual plate height; b) Horizontal references for changes in buccal and lingual width at 1, 3 and 5mm; c) and d) Superimposition of CBCT1 and CBCT2.

Horizontal dimensional changes were assessed by measuring the distance from the vertical reference line to the buccal and lingual bone plates at 1, 3 and 5mm apical to the most coronal aspect of the alveolar crest in both CBCT1 and CBCT2. Width differences between time points were calculated to determine horizontal bone dimensional changes at 4 months (Figure 2).

2Vertical bone height changes, defined as the primary outcome, were evaluated by measuring the distance from the buccal and palatal bone peaks to the horizontal reference line in both CBCT1 and CBCT2. Differences between baseline and follow-up measurements were calculated to determine vertical bone resorption at the buccal and palatal aspects.

Accordingly, the following measurements (in mm) were recorded:

- Buccal Width at 1mm (BW1): Buccal bone resorption 1mm below the alveolar crest.

- Buccal Width at 3mm (BW3): Buccal bone resorption 3mm below the alveolar crest.

- Buccal Width at 5mm (BW5): Buccal bone resorption 5mm below the alveolar crest.

- Lingual Width at 1mm (LW1): Lingual bone resorption 1mm below the alveolar crest.

- Lingual Width at 3mm (LW3): Lingual bone resorption 3mm below the alveolar crest.

- Lingual Width at 5mm (LW5): Lingual bone resorption 5mm below the alveolar crest.

In addition, volumetric bone changes (secondary outcome) were assessed by superimposition of STL models generated from baseline and 4-month CBCT datasets using 3D Slicer® software. Absolute differences between time points were calculated, and changes were also expressed as percentages.

Statistical analysis

Assumptions underlying the ANCOVA models were formally assessed for all outcome variables. Normality of residuals and homogeneity of regression slopes were confirmed for all models, with no significant violations detected.

All dependent variables were analyzed using analysis of covariance (ANCOVA) to compare dimensional changes between treatment groups. A classification and regression tree (CRT) model was applied to identify cut-off values of the bone phenotype variable associated with increased resorption. Measurement reliability was assessed using the intraclass correlation coefficient (ICC) for continuous variables, with 95% confidence intervals.

All statistical analyses were performed using SPSS software (version 28.0; IBM Corp., Chicago, IL, USA). The level of statistical significance was set at 5% (=0.05).

## Results

**Figure 3:**
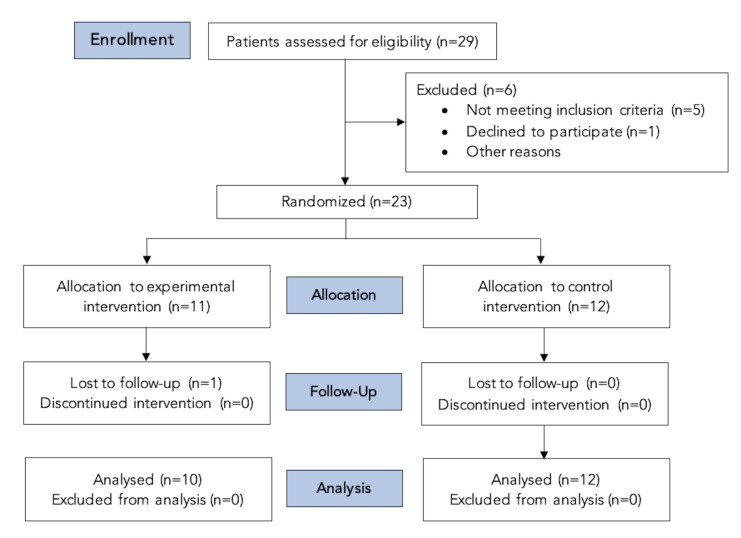
Flow chart of the study design and follow-up process.

Twenty-nine patients were initially screened for this randomized clinical trial. Of these, six were excluded (five patients did not meet the inclusion criteria and one patient declined to participate). Consequently, 23 patients were enrolled and allocated into two groups: 11 patients in the experimental group (mSST) and 12 patients in the control group (USH). One patient from the experimental group was lost to follow-up for personal reasons. Ultimately, 22 patients completed the study (10 men and 12 women), with a mean age of 55.15 years (Figure 3).

3All participants were systemically healthy, and no intraoperative or postoperative complications were recorded during the surgical procedures or the follow-up period. No statistically significant differences were observed between groups with respect to age, gender, smoking status, tooth position, reason for extraction, or baseline buccal bone thickness (BBT) (Table 1). Mean BBT values measured at 1, 3 and 5mm apical to the alveolar crest are also presented in Table 1.

Table 1Superimposition of CBCT datasets obtained at baseline and at 4 months postoperatively revealed significantly lower dimensional changes of the alveolar ridge in the mSST group compared with the USH group. Mean horizontal bone width reduction at 1, 3 and 5mm from the alveolar crest was 0.46, 0.33 and 0.70mm, respectively, in the mSST group, compared with 1.78 mm at 1 mm, 1.51mm at 3mm, and 1.24mm at 5mm in the control group.

Regarding vertical dimensional changes, mean buccal height reduction was 0.10mm and lingual height reduction was 0.52mm in the mSST group, compared with 1.63mm and 1.19mm, respectively, in the USH group. Although no statistically significant differences were detected between groups at the lingual aspect, lower dimensional changes were consistently observed in the mSST group across all evaluated outcomes (Table 2).

Table 2Statistically significant differences between groups were observed for buccal height reduction (1.527mm; 17.59%; p&lt;0.001), buccal bone width reduction at 1mm (0.982mm; 10.82%; p&lt;0.001), 3mm (0.783mm; 7.87%; p&lt;0.001), and 5mm (0.545mm; 5.85%; p&lt;0.001). In addition, a significant difference in volumetric bone resorption of 26.2mm³ was detected between groups (p&lt;0.001).

Table 3 presents the influence of baseline buccal bone thickness on buccal dimensional changes. In cases with a BBT &lt;1.5mm, statistically significant differences between groups were observed. In the control group, greater buccal bone resorption was found in sites with thinner BBT, whereas in the experimental group, resorption values were comparable regardless of baseline BBT. Within the control group, statistically significant differences between BBT categories were detected for buccal height (17.82% vs. 30.07%; p&lt;0.001), buccal width at 1mm (9.53% vs. 19.02%; p&lt;0.001), buccal width at 3mm (6.02% vs. 15.55%; p&lt;0.001), and buccal width at 5mm (4.69% vs. 10.10%; p&lt;0.001). No statistically significant differences were observed for vertical or horizontal bone resorption at the lingual aspect.

Table 3

## Discussion

The present randomized clinical trial evaluated the effect of preserving a thin buccal portion of the root using the modified socket shield technique (mSST) on alveolar ridge preservation (ARP), in comparison with unassisted socket healing (USH) following tooth extraction at non-molar sites. The results demonstrated significantly reduced buccal bone resorption in the mSST group, supporting the biological rationale underlying techniques aimed at preserving the periodontal ligament-bundle bone (PL-BB) complex.

Tooth extraction disrupts both the periodontal ligament and the bundle bone, a tooth-dependent structure that constitutes the main vascular supply to the facial bone wall, particularly in regions where the buccal plate is thin (18 - 20). This anatomical dependency explains the disproportionate resorption on the buccal aspect compared with the palatal or lingual side following extraction (21 , 22). Previous histological and clinical studies have reported up to 2.2mm of facial bone loss within the first eight weeks (2), with even greater losses occurring in thin-walled phenotypes, where up to 7.5mm of vertical collapse has been described (23). The results of the present study are consistent with these findings, showing more pronounced buccal alterations in the control group.

Preserving the buccal root fragment with mSST maintains the PL-BB complex, ensuring continuity of the vascular supply that would otherwise be lost during extraction. This preserved vascularization appears crucial for maintaining ridge stability. In addition, the avoidance of flap elevation, as performed in the present study, preserves periosteal blood flow, which is another important contributor to crestal stability (24 , 25). Therefore, the combination of preserving the buccal root fragment and performing atraumatic, flapless extractions provides a biologically advantageous environment for minimizing ridge resorption.

The present results are in agreement with previous clinical and radiological studies demonstrating the efficacy of the socket shield concept. De Oliveira et al. reported significantly reduced buccal-to-palatal crest resorption in SS-treated sites compared with fresh sockets (26). Similarly, a retrospective study using CBCT superimposition reported more stable facial and lingual bone levels in socket shield cases compared with immediate implant placement after five years of follow-up (27). More recently, Badawy et al., using the same evaluation protocol applied in the present study, reported significantly less reduction in buccal plate height and ridge width in the mSST group compared with the spontaneous healing group (28). Collectively, these findings reinforce the biological and clinical relevance of preserving the buccal root fragment.

Buccal bone thickness (BBT) played a significant role in the dimensional changes observed. Thin BBT is a well-recognized risk factor for severe post-extraction resorption,(29) particularly in the anterior maxilla, where most sites present &lt;1mm of buccal plate thickness. (30) In the present study, a threshold of 1.5mm was identified, below which mSST conferred a clear benefit. Sites with BBT &lt;1.5mm showed significantly greater preservation in the mSST group, whereas sites with BBT &gt;1.5mm did not show substantial differences between groups. These findings suggest that mSST may be especially beneficial in thin bone phenotypes, while in thick-walled phenotypes, atraumatic flapless extraction alone may be sufficient to maintain ridge dimensions.

The modified socket-shield technique is a technically demanding and operator-dependent procedure. A significant learning curve has been described, particularly with respect to root sectioning, preservation of the buccal fragment, and avoidance of micromovement or fracture of the shield (27). Inadequate execution may result in complications such as partial or complete shield mobilization, infection, exposure of the retained fragment, or unintended damage to the buccal plate (12). These complications have been primarily associated with inadequate case selection and limited surgical experience; therefore, the present findings should be interpreted with caution, as outcomes achieved under controlled conditions may not be directly extrapolated to routine clinical practice.

Despite the favorable outcomes, several methodological limitations should be acknowledged. The follow-up period was limited to 4 months, which does not allow assessment of the long-term stability of the observed dimensional changes; however, this time point was intentionally selected to capture the early phase of post-extraction bone remodeling. The sample size calculation was performed exclusively for the primary outcome variable, and although the final sample exceeded the minimum required per group, the study may have been underpowered to detect small or moderate effects in secondary or exploratory analyses. Furthermore, clinical and aesthetic soft tissue parameters were not evaluated, as the study was designed to specifically isolate hard tissue dimensional changes using standardized radiographic and volumetric methodologies. Consequently, secondary outcomes should be interpreted with caution. Future investigations incorporating longer follow-up periods, larger cohorts, and combined hard and soft tissue assessments are required to confirm these findings.

Overall, the findings of this study support the biological rationale and clinical utility of mSST for ARP, particularly in sites with thin buccal plates. Preservation of the PL-BB complex appears to be fundamental in minimizing post-extraction dimensional changes, reinforcing the relevance of the socket shield concept in contemporary implant and ridge preservation strategies.

## Conclusions

The modified socket shield technique (mSST) demonstrated a protective effect against post-extraction alveolar bone remodeling when compared with unassisted socket healing. Preservation of the buccal root fragment resulted in significantly reduced horizontal and vertical dimensional changes at the buccal aspect, particularly in sites presenting a thin buccal bone phenotype (&lt;1.5mm).

## Figures and Tables

**Table 1:** Table Demographic data of the included patients.

Baseline characteristics	Control Group	Experimental Group
Age (years)	59.83 (8.54)	50.5 (8.92)
Male/Female	5/7	5/5
Smokers	2	2
Maxilla/Mandible	8/4	7/3
Incisors/Canines/Premolars	5/1/6	4/1/4
Reason for extraction(caries/endodontic failure/fracture/prosthetic reasons)	5/2/2/3	4/1/1/2
BBT 1mm below crest	0.94±0.50mm	1.06±0.51mm
BBT 3mm below crest	1.02±0.47mm	1.18±0.55mm
BBT 5mm below crest	1.21±0.41mm	1.346±0.59mm
BBT > 1,5mm/<1,5mm	3/9	3/7

BBT: Buccal bone thickness.

**Table 2:** Table Mean dimensional changes between the baseline and the 4 months follow-up.

Outcome	Group	N	Mean mm (%)	SD	Medians	Significancea
BW1	Test	10	0.069 (0.98±0.92)	0.06	0.06	<0.001
	Control	12	1.051 (11.80±8.69)	0.70	0.93	
BW3	Test	10	0.067 (1.24±1.28)	0.12	0.08	<0.001
	Control	12	0.850 (9.11±7.08)	0.58	0.64	
BW5	Test	10	0.058 (0.66±0.67)	0.05	0.04	<0.001
	Control	12	0.603 (6.51±4.37)	0.36	0.50	
LW1	Test	10	0.392 (4.82±4.66)	0.33	0.32	0.381
	Control	12	0.733 (8.21±8.53)	0.75	0.40	
LW3	Test	10	0.269 (3.07±2.89)	0.25	0.21	0.314
	Control	12	0.661 (7.55±7.89)	0.82	0.35	
LW5	Test	10	0.335 (3.54±3.03)	0.32	0.21	0.228
	Control	12	0.642 (7.11±7.21)	0.72	0.50	
BH	Test	10	0.103 (1.01%±0.58)	0.06	0.08	<0.001
	Control	12	1.630 (18.60±17.13)	1.27	1.18	
LH	Test	10	0.512 (5.29±3.68)	0.38	0.38	0.050
	Control	12	1.198 (13.64±13.26)	1.01	0.86	
Volume	Test	10	6.23mm3	2.43	6.57	<0.001
	Control	12	32.43mm3	1.41	26.52	

SD: Standard deviation. a: The significance level is 0.050. BW1: Buccal bone width at 1mm. BW3: Buccal bone width at 3mm. BW5: Buccal bone width at 5mm. LW1: Lingual/Palatal bone width at 1mm. LW3: Lingual/Palatal bone width at 3mm. LW5: Lingual/Palatal bone width at 5mm. BH: Buccal height. LH: Lingual height.

**Table 3:** Table Buccal bone thickness influence in the mean dimensional changes between baseline and the 4 months follow-up.

Outcome	BBT (mm)	Control group	Test group
BW1	<1.5	16.02%*	0.58%
	>1.5	9.53%	0.58%
BW3	<1.5	14.55%*	1.73%
	>1.5	6.02%	1.09%
BW5	<1.5	9.10%*	1.01%
	>1.5	4.69%	0.92%
BH	<1.5	9.82%*	1.64%
	>1.5	20.07%	0.63%

a: The significance level was 0,050. BW1: Buccal bone width at 1mm. BW3: Buccal bone width at 3mm. BW5: Buccal bone width at 5mm. BH: Buccal height. *significance (<0.05).

## Data Availability

Declared none.
